# Dr. Mary Putnam Jacobi (1842-1906): The Pioneer of Women’s Medical Education

**DOI:** 10.7759/cureus.69081

**Published:** 2024-09-10

**Authors:** Katherine N Girgis, Latha Ganti

**Affiliations:** 1 Research, Orlando College of Osteopathic Medicine, Winter Garden, USA; 2 Emergency Medicine & Neurology, University of Central Florida, Orlando, USA; 3 Medical Science, The Warren Alpert Medical School of Brown University, Providence, USA

**Keywords:** mary putnam jacobi, medical education, menstruation myths, publications, women's medical association

## Abstract

Mary Putnam Jacobi (1842-1906) was an integral figure in the fight for women’s rights to medical education and a pioneer in the debunking of prevailing menstruation myths. Her experiences being denied adequate medical education in the United States led her to pursue medical education in Paris, where she learned the importance of laboratory experimentation and was first introduced to the political discourse surrounding women’s rights. She founded the Women’s Medical Association and created a medical curriculum for women that paralleled the standards of Harvard Medical School. Her research publications proved that women could conduct work with the same rigor as men and advocated for women’s integration into workplaces and educational spaces exclusive to men. Mary Putnam Jacobi’s contributions to the women’s suffrage movement created new opportunities for women to attain high levels of education that otherwise would not exist today and continue to inspire women to push the limits placed on them solely due to their gender.

## Introduction and background

Early life

Mary Putnam Jacobi was born on August 21, 1842, in London, United Kingdom. Her family returned to the United States in 1848, and she was raised as the eldest of 11 in Staten Island, New York. She was primarily educated by her mother at home and later attended a private school in Yonkers. From a young age, her parents encouraged her to pursue a literary vocation, and she often contemplated the meaning of life in her childhood letters. However, throughout her formative years, she observed ample amounts of human suffering for the duration of the Civil War. This experience shifted her interests toward a more curative profession [1].

**Figure 1:**
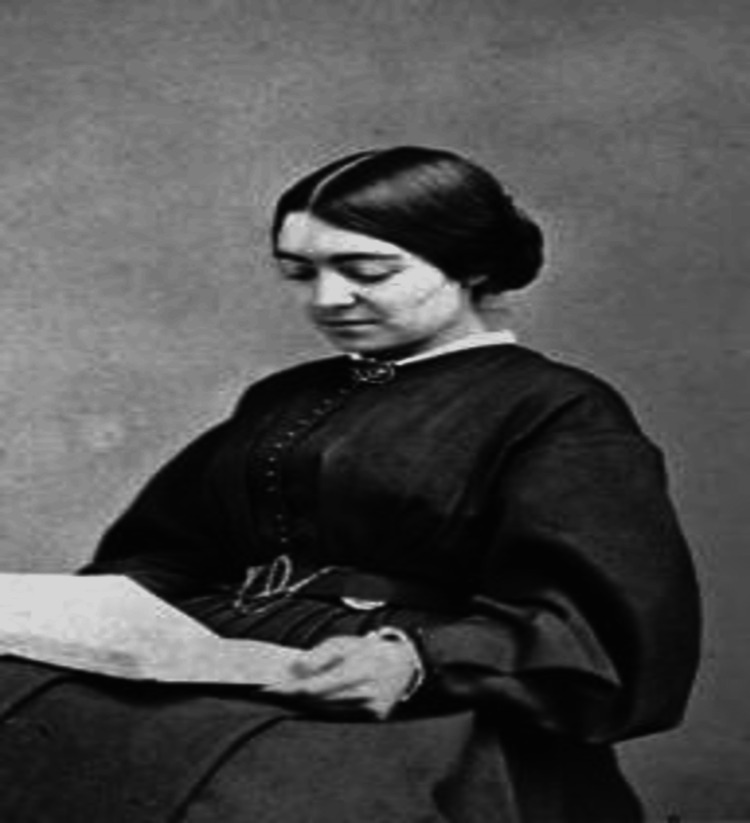
Mary Putnam Jacobi as a medical student (photograph by Bogardus). Credit: The Schlesinger Library, Radcliffe Institute. From WikimediaCommons; image in public domain with no known copyright restrictions

After her graduation in 1859, her decision to pursue medicine was not well received by her family. It is important to note that, during the 1860s, the idea of a woman pursuing a professional career was difficult for parents to support. Even with Mary’s advanced chemical education, it was still expected that she take up the domestic obligations associated with being a woman and the eldest of a large family. Her father was wary of her pursuit, denoting the career as “repulsive” for a woman, and wrote letters encouraging her to delay it a number of years in favor of aiding her mother with child-rearing [2] (Figure 1).

Pursuit of medical education

Mary Putnam Jacobi’s foray into the medical career began with her degree from the Female Medical College of Pennsylvania in 1864. Her initial degree in pharmacology led her to an internship at the New England Hospital; however, she still desired further medical education at a standard that was not attainable by women in the United States at the time. The limitations on women’s right to education during this period implored her to broaden her search to schools outside of the country. In 1866, she became the first woman to be admitted to the Paris l’École de Médecine, a feat described as “a Paris medical degree and a training in scientific medicine unusual at that date even among men” [3].

The education Jacobi received in Paris provided her with opportunities to participate in the medical laboratory. The hands-on experiences she cultivated through the dissection of animal organs and examination of histology and cellular pathology deeply altered her thought process. It proved to be a foundational aspect of her further research, as she believed all expertise should be based on observable circumstances [1]. During her Parisian education, she witnessed the Franco-Prussian War and had frequent contact with French proponents of social change and women’s rights. These encounters formed the basis for her arguments for experimentalism and advocacy for women’s suffrage [1].

## Review

Fight for women’s suffrage

It was through scientific knowledge and the profession of medicine that Mary Putnam Jacobi began to advocate for women’s rights in American society. She believed that full equality and independence for women could only be achieved by utilizing scientific knowledge of the female body to justify womens’ position in higher education and professional roles. This unique approach directly intertwined politics with the practice of medicine itself. Furthermore, this approach garnered the respect of many medical men, allowing her to gain entry into associations that previously barred women from participating, paving the way for future medical women to engage in medical discourse [4].

Women who managed to attain a medical education during the 1880s were often graduates of homeopathic schools, which focused on training them in midwifery, particularly gearing toward the care of children. Women’s role in medicine was based on the idea that they were a softer, more compassionate gender, which was suited to sympathetic care. It was Jacobi who gave lectures denouncing this philosophy, believing that this was a viewpoint that undermined women’s ability to follow a scientific and fact-based model of medicine. She renounced the idea that science and logic were masculine domains and advocated for the inclusion of women in laboratory spaces [1].

Establishing a women’s society

In 1878, the discrepancies in medical education between men and women were still based on the idea that men were fundamentally more scientific than women. To address this disparity, Mary Putnam Jacobi founded and served as president of the Association for the Advancement of Medical Education of Women. She argued that women interested in the pursuit of medicine were crippled by organizational opposition and that women could not be considered inferior physicians to men unless they were first provided the same standard of education. Under this association, she expanded the curriculum of the Woman’s Medical College of the New York Infirmary to include extensive laboratory dissections and anatomical observation [3]. Through these endeavors, this women’s medical institute quickly surpassed the curriculum of many men’s medical colleges and soon became the only school to match the standard of Harvard Medical School [1,3]. The association was later renamed the Women’s Medical Association and continued to pursue equal educational opportunities for women.

Pioneering research on menstruation and hysteria

During the 1870s, prevailing research from Harvard faculty on menstruation proposed that women were impaired by the process and required rest throughout the duration of the cycle [5]. Mary Putnam Jacobi’s 1876 publication "The Question of Rest for Women During Menstruation" was initially penned under a pseudonym, as medical publications at this time would reject research conducted by women. In this book, Jacobi applied the scientific method to the study of women’s bodies in a more comprehensive view than any prior research. Of the 268 women Jacobi surveyed on experiences during menstruation, over one-third of the responses indicated that they were not conscious of any change throughout the menstrual cycle [6]. Her study proved that rest did not prevent menstrual pain and argued that the call for bed rest was simply a rationale to prevent women from advancing in the workplace, perpetuating male superiority in work positions and exclusivity in educational institutions [5,7]. The study garnered significant praise and was awarded the 1876 Boylston Prize by Harvard University. When the institution presented the award to Mary Putnam Jacobi, they were outraged at the discovery that the revolutionary study was spearheaded by a woman [7]. Her work proved that women could present logical and scientific data, a domain once thought to be exclusively male, and equally compete with men in the field of medicine.

Mary Putnam Jacobi’s later works delved into the prevalence of hysteria among women and its etiology. In her 1888 volume "Essays on Hysteria," she argued that hysteria was a disease of nutritional inadequacy, linking it to lack of nerve stimulation. She proposed that to prevent and treat hysteria, social and economic conditions for women should be improved to allow them more active roles in academia and workplaces. Jacobi’s direct linkage of hysteria to inadequate neural stimulation further advocated for women’s rights to emancipation and pushed for their presence in male-dominated spaces [8].

Modern impact

Mary Putnam Jacobi believed that the advancement of women in medicine would profoundly transform the daily lives of ordinary women in American society. She hoped for a future where most middle-class women would not only have basic scientific knowledge but would also find frequent applications for it in their daily lives. Largely due to her efforts, Johns Hopkins Medical School finally opened its doors to women [3]. Women are provided the same access to medical education as men and are given equal opportunities to pursue laboratory experience. Furthermore, the belief that women are entirely debilitated by the process of menstruation is nothing more than a myth [1].

## Conclusions

Mary Putnam Jacobi’s commitment to women’s suffrage is a major contributor to the freedom that modern-day women experience. Her work in the Women’s Medical Association achieved medical educational standards for women that rivaled Harvard’s, proving that women were just as capable of scientific experimentation as men. Furthermore, her disproval of the myth that women require rest during menstruation demonstrated that women could maintain the same work ethic as their male counterparts. Her research on hysteria further advocated for the advancement of women in work positions, as she linked its etiology directly to a lack of neural stimulation. Jacobi’s legacy provided a gateway for women into the world of medicine and, as a result, improved the lives of countless women and patients who would otherwise lack access to this care.
